# Developing mHealth Messages to Promote Postmenstrual Regulation Contraceptive Use in Bangladesh: Participatory Interview Study

**DOI:** 10.2196/mhealth.6969

**Published:** 2017-12-14

**Authors:** Elisabeth Eckersberger, Erin Pearson, Kathryn Andersen, Altaf Hossain, Katharine Footman, Kamal Kanti Biswas, Sadid Nuremowla, Kate Reiss

**Affiliations:** ^1^ Ipas Chapel Hill, NC United States; ^2^ Marie Stopes International London United Kingdom; ^3^ Harvard TH Chan School of Public Health Boston, MA United States; ^4^ Association for Prevention of Septic Abortion, Bangladesh Dhaka Bangladesh; ^5^ Ipas Bangladesh Dhaka Bangladesh; ^6^ Marie Stopes Bangladesh (at the time of the study; currently: Independent Consultant) Dhaka Bangladesh; ^7^ London School of Hygiene & Tropical Medicine London United Kingdom

**Keywords:** abortion, reproductive health services, contraception, family planning, mHealth, Bangladesh

## Abstract

**Background:**

Abortions are restricted in Bangladesh, but menstrual regulation is an approved alternative, defined as a procedure of regulating the menstrual cycle when menstruation is absent for a short duration. Use of contraception after menstrual regulation can reduce subsequent unintended pregnancy, but in Bangladesh, the contraceptive method mix is dominated by short-term methods, which have higher discontinuation and failure rates. Mobile phones are a channel via which menstrual regulation clients could be offered contraceptive support after leaving the clinic.

**Objective:**

This study aimed to support the development of a mobile phone intervention to support postmenstrual regulation family planning use in Bangladesh. It explored what family planning information women want to receive after having a menstrual regulation procedure, whether they would like to receive this information via their mobile phone, and if so, what their preferences are for the way in which it is delivered.

**Methods:**

We conducted participatory interviews with 24 menstrual regulation clients in Dhaka and Sylhet divisions in Bangladesh. Women were recruited from facilities in urban and peri-urban areas, which included public sector clinics supported by Ipas, an international nongovernmental organization (NGO), and NGO clinics run by Marie Stopes. Main themes covered in the interviews were factors affecting the use of contraception, what information and support women want after their menstrual regulation procedure, how respondents would prefer to receive information about contraception, and other key issues for mobile health (mHealth) interventions, such as language and privacy. As part of the in-depth interviews, women were shown and played 6 different messages about contraception on the research assistant’s phone, which they were given to operate, and were then asked to give feedback.

**Results:**

Women were open to both receiving messages about family planning methods on their mobile phones and talking to a counselor about family planning methods over the phone after their menstrual regulation. Women most commonly wanted information about the contraceptive method they were currently using and wanted this information to be tailored to their particular needs. Women preferred voice messages to text and liked the interactive voice message format. When asked to repeat and identify the main points of the messages, women demonstrated good understanding of the content. Women did not seem too concerned with privacy or with others reading the messages and welcomed including their husbands in speaking to a counselor.

**Conclusions:**

This study found that menstrual regulation clients are very interested in receiving information on their phones to support family planning use and wanted more information about the method of contraception they were using. Participatory voicemail was the preferred modality.

## Introduction

### Background

Rapid expansion in mobile phone ownership has led to the development of numerous mobile health (mHealth) interventions targeting a wide range of health issues in both developed and developing countries [1,2]. This relatively new field of public health has the advantage of being able to reach large audiences with targeted, personalized information at low cost, including individuals who may not have easy access to health services. In 2015, there were 82 phone subscriptions for every 100 inhabitants in Bangladesh [3], and by 2012, two-thirds of rural women of reproductive age reported that their household owned at least one functional mobile phone [4]. mHealth has been used successfully in Bangladesh to facilitate identification and treatment of malaria [5], provide timely care for obstetric emergencies [6], improve vaccination rates [7], and enable glycemic control among patients with type 2 diabetes [8].

Abortions are legal only to save a woman’s life in Bangladesh, but menstrual regulation is an approved procedure, defined as regulating the menstrual cycle when menstruation is absent for a short duration [9] up to 12 weeks after their last period [10]. Menstrual regulation services use manual vacuum aspiration (MVA) or the combination regimen of mifepristone and misoprostol to establish nonpregnancy, and pregnancy is not confirmed before the procedure or administration of medications. Menstrual regulation procedures are common in Bangladesh and occur at a rate of 37 per 1000 women of reproductive age [11].

Provision of family planning services to menstrual regulation clients offers an important opportunity to increase uptake of effective contraception and reduce the need for repeated menstrual regulation services, as this population often wishes to delay or limit future pregnancies. A global systematic review found that the existing evidence on the effectiveness of postabortion family planning counseling and services in low-income countries to address the problem of unsafe abortion is inconclusive; studies showed increases in contraceptive uptake, but none provided evidence on its effectiveness on maternal morbidity and mortality [12]. In Bangladesh, menstrual regulation guidelines recommend the provision of family planning services, yet in one study fewer than half of facilities offered contraceptive methods to postmenstrual regulation clients, and fewer than half of these ultimately received a method [13]. Where there is postmenstrual regulation counseling, it is generally conducted by either designated counselors or family planning staff; providers typically offer a range of family planning methods: oral contraceptive pills, injectables, and intrauterine contraceptive device (IUCD). Implants are offered in some locations, and condoms are sometimes suggested to clients who do not wish to take other family planning methods (personal communication with Bangladeshi family planning expert). Quality of care, including the quality of counseling and availability of family planning methods, varies widely [14]. These inequalities in postmenstrual regulation family planning services have been shown to impact contraceptive use: nearly all women who received the highest quality of care were using a modern method of contraceptive 3 months post procedure, whereas rates for those who received the lowest quality care were significantly lower [14]. The type of contraception women use also has implications for how successfully they can prevent unintended pregnancy. Use of short-term methods of contraception is common in Bangladesh [15]; however, short-term methods (condoms, pills and injectable) are less effective at preventing pregnancy than long-acting methods (implant, IUCD and sterilisation) and have higher rates of discontinuation [15].

Regardless of quality of care and counseling, some women may not want to make a decision about family planning on the day of the procedure [16]. mHealth offers an opportunity to reach women with information about family planning after they have left the clinic; however, evidence for the effectiveness of mobile phones in increasing contraceptive use is limited and mixed [17]. A recent Cochrane review found just 5 high-quality studies, and only 1 of these focused on postabortion populations; a randomized controlled trial (RCT) of mobile phone voice messages to support postabortion contraception in Cambodia found an increase in long-acting contraceptive use at 12 months among the intervention group [17,18].

Studies investigating the feasibility of mHealth interventions in Bangladesh found a number of barriers, including inadequate understanding of how mobile phones work [19] and the fact that most mobile phones lack the capacity to show and type in Bengali script [20,21]. An evaluation of an mHealth intervention to support contraceptive knowledge in Kenya found similar concerns; lack of English literacy is seen as a possible reason for the skewed demographic profile of intervention users [22].

Recent work in Bangladesh on the adoption of mHealth based on the extended technology acceptance model [23] found that perceived ease of use, perceived usefulness of the intervention, and subjective norms all had significant positive impact on the intention to adopt mHealth services, although personal innovativeness (the desire to be among those first to adopt a new technology) in information technology was not significant. Formative research and tailoring of interventions to specific settings and groups are important to ensure adoption but are often omitted; a systematic review of 44 papers on mHealth behavior change communication interventions found that less than half of the reviewed interventions described targeting or tailoring the content [2].

### Objective

This study aimed to inform the development of a mobile phone intervention to support postmenstrual regulation family planning use and decision making by exploring what family planning information women in Bangladesh want to receive after having an menstrual regulation procedure, whether they would like to receive this information via their mobile phone, and if so, what their preferences are for the way in which it is delivered. This formative research has been used to design an intervention, which is being evaluated by an RCT, and results are forthcoming.

## Methods

### In-Depth Interviews

Between March and June 2015, we conducted 24 participatory in-depth interviews with menstrual regulation clients from 7 facilities located in Dhaka and Sylhet divisions in Bangladesh. Facilities were selected from urban and peri-urban areas with varying socioeconomic surroundings, with the aim of accessing the views of women living in those particular areas. The facilities included public sector clinics supported by Ipas, an international nongovernmental organization (NGO), and NGO clinics run by Marie Stopes. We planned to conduct 32 interviews across 8 facilities, but one relatively rural facility was dropped because it was inaccessible during the data collection period because of political violence. To maintain our timeline, 24 women sampled from 7 facilities were interviewed.

A convenience sample of women was recruited from the selected facilities. Women were eligible to participate if they were aged between 18 and 49 years, had received menstrual regulation services using MVA or medication from the facility, had not received general anesthesia for their procedure, and had a personal mobile telephone with short message service (SMS) capability. The menstrual regulation provider introduced clients to the research assistant after the completion of their menstrual regulation procedure and postprocedure counseling. The research assistant told the client about the study, determined whether she was eligible to complete the interview, administered informed consent, and conducted the interview. Research assistants were nonclinic staff, which gave women the opportunity to speak more freely about their experiences at the clinic. A total of 4 female research assistants received 3 days of training, which included familiarization with the study topics, objectives, and interview guide and role-play practice sessions with feedback. The importance of informed consent was stressed.

The research assistants gave women the option to complete the interview the same day or within 2 days of their procedure; women could complete the interview at the clinic or could agree to a different location with the research assistant. All women gave written informed consent before the interview was conducted.

### Development and Content of the In-Depth Interviews

We developed the in-depth interview guide in English, and then translated it into Bengali. We pilot-tested the interview guide through peer testing and further adapted it during the interviewer training. The main themes covered in the interviews were factors affecting the use of contraception, what information/support women want after their menstrual regulation procedure, how respondents prefer to receive information about contraception, and other key issues for mHealth interventions, such as language and privacy.

As part of the in-depth interviews, women were shown and played 6 different messages about contraception on the research assistant’s phone, which they were given to operate (see Multimedia Appendix 1 for interactive voice message format), and were then asked to give feedback on these. The interactive voice message did not connect to a call center, but included this as an option to illustrate to participants how the system could work. The messages shared in the interviews were adapted from a series of messages developed and validated by FHI 360, a nonprofit organization based in the United States that aims aimed to support contraceptive use using mobile phones [22,24].

After hearing and seeing the messages, respondents discussed what type of modality they preferred (SMS or voice messages, interactive or one-way messages), what they thought of different styles and genres (eg, reminders, factual information, or personal stories), and what kind of information they would want to receive.

Of the 6 messages that were shared in the interview, 2 were brief reminder messages, one about oral contraceptive pills and the other about injections; 2 were factual messages about the effectiveness and side effects of the IUCD, respectively; and 2 messages were stories about women’s experience of using contraceptives, one about using pills and the other on the IUCD (see Textbox 1). To test different modalities, the IUCD message was sent as a text SMS, a voice message, and as part of a interactive voice message with additional options to select at the end of the message. The SMS message was written in phonetic Bengali in English letters, as most phones do not have the capability to type or show Bengali script. For the one-way voice message, an automated message played after the phone was picked up. The interactive voice message worked in the same way but, at the end of the message, the caller could choose from a number of options by pressing a number on the keypad.

We tape-recorded interviews and then transcribed and translated them into English. We used qualitative thematic analysis; we created codes based on themes both from the interview guide and from concepts that emerged from the data. We created the codebook and final themes in an iterative process. We presented initial results at a workshop in Dhaka, and we included comments from the authors and workshop participants in additional analysis and synthesis. All coding was done using nVivo 10 (QSR International Pty Ltd, London, UK).

We received ethical approval from the Bangladesh Medical Research Council, the Population Council Institutional Review Board, and the Marie Stopes International Independent Ethical Review Committee.

**Content of messages shared in the in-depth interviews.**

Messages 1 and 2: Pill and injection use (Reminder)

- Remember to take your pill.
- Your dose is due next week. You can return to the clinic to get your next dose. 

Message 3: IUCD effectiveness (Factual)

- IUCD is a small device placed inside the womb. Highly effective for 5 to 12 years. May increase monthly bleeding and cramps at first. When removed, can become pregnant with no delay. No infertility or birth defects; does not move around in body.

Message 4: IUCD side effects (Factual)

- IUCD has few side effects. Monthly bleeding may be irregular, heavy, and longer during the first 3 to 6 months. May have painful cramps. Wounding of the uterus is rare. Some women don’t have side effects.

Message 5: Pill use (Story)

- I called my sister Sumi and she told me she used oral contraceptive pills (daily pills) for 2 years after she married Mahmud. She stopped to start a family. Now Sumi and Mahmud have 2 beautiful children. Sumi said that lately Mahmud is worried about the cost of raising 2 kids, so she is using daily pills again and now they don’t worry about unplanned pregnancy. Sumi uses the alarm on her mobile as a reminder to take her pill every night before bed. Mahmud makes sure Sumi has new pill packs when she needs them. What a great guy—I wonder if my husband would do that for me?

Message 6: IUCD use (Story)

- After Samreen got married to Rifat, she knew she didn’t want to get pregnant for a few years, but she wanted to use a method that she didn’t have to think about. Her sister-in-law Maliha used an IUCD for 2 years before she had her first son Shehan. She recommended that Samreen get one, too. Samreen was a bit nervous about having it fitted, but she’s happy she did. She doesn’t have to worry about getting pregnant, and she doesn’t have to remember to do anything either.

## Results

In total, 24 in-depth interviews were completed with women aged between 18 and 34 years, with an average age of 25 years. All women were married, were ethnically Bengali, and had undergone MVA procedures. Sample characteristics (Table 1) show that we possibly oversampled urban women and those with higher levels of education.

### Overall Response to the Idea of Receiving Family Planning Messages

Women were overwhelmingly open to both receiving messages about family planning methods on their mobile phones and talking to a counselor about family planning methods over the phone after their menstrual regulation. Women generally felt that a female counselor would be better, but many reported that they would not have a problem speaking to a male counselor.

Women were asked whether they would prefer free messages or whether they would be willing to pay for them, and although they generally felt that a free service would be preferable, many said they were willing to pay if necessary. It seemed reasonable to pay for information you needed, and it was also suggested that people would pay more attention to the messages if they had to pay for them:

It is better to have calls free of cost. But if they demand money for this call, I am ready to pay it.

Participant 8

Bengali people don’t pay much attention towards free things. They pay much more attention towards paid benefits.

Participant 4

### What Information Do Women Want?

When women were asked what information about family planning they would like to receive after their menstrual regulation, the most common responses were to have content about the contraceptive methods they had selected after their menstrual regulation procedure as well as overall advice on family planning methods. Many women were interested in receiving more information about what methods would “suit” them, suggesting interest in information tailored to them and their situation:

I would also like to know about the method that will suit my body.

Participant 9

When asked what kind of messages they would want after a menstrual regulation procedure, women wanted to know specifically about what family planning methods they could use at this time. Women also had menstrual regulation related questions about returning to work, how long to refrain from intercourse, what medication they should be taking, and their future fertility.

**Table 1:** Sample characteristics.

Participant	Age	Currently employed	Highest education	Children	Division	Urban/rural
1	20	No	Primary	1-2	Sylhet	Peri-urban
2	26	Garment sector	Secondary	1-2	Dhaka	Urban
3	30	No	Primary incomplete	1-2	Dhaka	Urban
4	28	Community Clinic	College	1-2	Dhaka	Urban
5	34	Health worker	Primary	3 or more	Dhaka	Urban
6	18	No	Primary	N/A^a^	Sylhet	Peri-urban
7	30	No	None	3 or more	Sylhet	Peri-urban
8	23	Herbal company	Primary incomplete	1-2	Dhaka	Urban
9	25	No	Secondary	1-2	Sylhet	Peri-urban
10	22	No	College	1-2	Dhaka	Urban
11	26	No	College	1-2	Dhaka	Urban
12	25	Garment sector	Primary	1-2	Dhaka	Urban
13	22	No	Primary	1-2	Dhaka	Urban
14	20	No	College	1-2	Dhaka	Urban
15	21	No	Primary	None	Dhaka	Peri-urban
16	21	No	College	None	Dhaka	Peri-urban
17	25	No	Secondary	1-2	Dhaka	Urban
18	25	No	Secondary	1-2	Dhaka	Urban
19	32	Nurse	Secondary	1-2	Dhaka	Urban
20	30	No	Secondary	None	Dhaka	Urban
21	24	Domestic worker	Primary	1-2	Dhaka	Urban
22	24	No	Primary	1-2	Dhaka	Peri-urban
23	34	No	None	3 or more	Sylhet	Peri-urban
24	22	No	Secondary	1-2	Sylhet	Peri-urban

^a^N/A: data not available.

Women spoke repeatedly about only wanting information that was relevant to them; they were not interested in hearing about alternative family planning methods. Participant 21, who is currently using condoms, said the following with respect to Message 3 (IUCD effectiveness):

Interviewer: Do you think that this is valuable message?

Respondent: Since I don’t use it, not to me.

Interviewer: Why?

Respondent: Like I said, it’s not important to me. I don’t use it, it probably is important to other people, but not to me.

Participant 21

Conversely, women also seemed to value new information and stated that knowledge about family planning methods was very important to them. Participant 19, currently not using an IUCD, said the following about Message 4 (IUCD side effects):

Interviewer : Was this message helpful?

Respondent: Yes.

Interviewer : How?

Respondent: Because I got to know new information, and I will let other people know about it as well.

Interviewer: How does the message meet your needs?

Respondent: It gave me a lot of new information.

Participant 19

### Technology and Language

Women generally preferred voice messages to SMS; the consensus was that reading SMS messages could be difficult or impossible for some women:

Those who are illiterate they will not be able to read. So, SMS is not convenient to them. For them voice mail is easy to understand.

Participant 7

They overwhelmingly said they preferred Bengali script and found the English hard to read and thought others would too:

If it had been in Bengali, the message would have been clearer to me.

Participant 1, via SMS

Women generally liked the interactive voice message and found this easy to understand. They also liked the different options this modality gave them and the fact that they could go straight to the information they wanted to hear:

I like the fact that I can press a button and get to know what I want to know; I don’t have to go through unnecessary information to get what I want to know.

Participant 17

Only one woman found any difficulty using the interactive voice message option:

Since I don’t have education it’s tough for me.

Participant 3

Women were however aware of the transitory nature of voice message—that it could not be accessed in their own time or recalled in the same way an SMS could. They liked the fact that the SMS came to their phones without actions on their part, even if the phone was switched off, and that they could read and reread in their own time, which they could not do with a voice message:

...if this message comes in my mobile just like an SMS, I can even read or see it later on, after finishing my job.

Participant 14

Something women mentioned throughout the interview, often unbidden and in response to different questions, was that they wanted a “fixed number” to call; they wanted somewhere they could ask their own questions in their own time:

Interviewer: Are you interested in subscribing for the number of the call center via SMS? Why?

Respondent: Yes, because if I need to know something else other than what they are providing in the voicemail service, I could ask.

Participant 16

Overall, women preferred messages that included voice message options because it would be accessible to women who are illiterate and may not be able to read SMS.

### Message Style

Overwhelmingly, women had no problem understanding the content of the messages. When relaying the meaning of messages, women differed in the sections they highlighted, possibly reflecting those aspects that they found most interesting or had concerns about, as examples of the responses to Message 3 (IUCD effectiveness) show:

For people who use the IUCD, if removed they will get pregnant. That is the basic message here.

Participant 11

What I understood from this message is that it remains in the same place in the uterus where it is placed.

Participant 23

...yes, it speaks about the validity of the IUCD for 5-12 years.

Participant 10

There were some instances, particularly concerning the IUCD, where women reported content that wasn’t included in the message, possibly reflecting prior knowledge, misconceptions, or concerns. When asked what they knew about the IUCD, women often cited concerns and worries before any factual aspects of the message, such as participant 5 speaking about Message 4 (IUCD side effects):

The main substance of the message is that many people are afraid of it while the others are not.

Participant 5

One woman referred to the fact that the IUCD is not popular and pointed out that stating side effects may make other women even more afraid (Message 4: IUCD side effects):

IUCD has some problems. If the message points out on the pain that may be felt during the implantation of it, many people will get afraid of it. Therefore, many people may ignore this message.

Participant 9

Women appeared to be somewhat torn between short, factual messages and longer story messages. They reacted strongly and emotionally to the messages portraying stories about families but at the same time reporting that these messages were too long and not informative enough.

Story messages were overall more emotion than content driven, and this is what women remarked on after hearing the messages. They also reported emotionally driven understanding that was different from the actual content of the message:

Now the husband had fear. But when it was given to her, he became worry free.

Participant 2, responding to Message 6

Family members are angry about her taking the pills.

Participant 13, responding to Message 5

Women however also found the story messages too long and not factual enough:

You need to mention about the topic of the message first. You need to make people understand about what you want to convey with this message. Only then they will read it.

Participant 17, responding to Message 5

Things can be removed and shortened. Most people don’t have the time to listen to such a big message.

Participant 18, responding to Message 6

Reminder messages (Messages 1 and 2: Pill and injection use) about short-term methods were well received and understood. Women said the reminder messages would be very useful to them, as it would remind them if they forgot to take the pill when they were busy with work and would remind women of the date of their next contraceptive injection, which they felt could be difficult to remember for rural women.

...reminding you is the biggest help that it does. In family planning mistakes only happen when you forget.

Participant 18

Women also wanted specifics in their reminders, to add specific time and date. In this case, to Message 2 (reminder about injection):

The later part of the message is okay “To take your next dose please report to the clinic.” But the first part needs the addition of date. If you can add the date, it would be more suitable. Other than that, if you don’t include the date, you can include the day of the week, which can work wonders too. I only say this because women from villages don’t remember dates well. If you mention the day of the week it would be great, for example “You need to take your injection dose by Thursday of the next week. To take your next dose please report to the clinic.”

Participant 4

### Privacy

Women were asked about privacy and whether it would be a problem if anyone saw or heard the messages. They generally reported that privacy would not be an issue. When asked specifically about which people should not see the message, in-laws and children were most commonly mentioned by participants who explained that this could cause embarrassment and shame:

Suppose, there are many senior father-in-laws or children. It becomes a matter of shame if they listen to it.

Participant 8

Women often responded to questions about privacy with answers welcoming sharing the message, so that others, including family members, could learn from it:

It is important because it is informing people who don’t know about it. In my case, I didn’t know about it much, so I got to know as well, and it is also reminding me of things I have learned in the past and forgot. When all of it will come to me, I will be able to explain it to other people.

Participant 19

### Husband’s Involvement

Women were asked whether they thought their husbands would want to talk to counselors over the phone. They felt their husbands would be interested to know more about family planning methods, that they could learn from the counselor, and that this would make them more helpful and supportive.

Women reacted very strongly to Message 5, the story message about the husband helping out with pills. They were very keen for their husbands to listen to and learn from this and to become more involved. The story’s protagonist’s relationship with her husband was by far the most remarked upon aspect of this message, and women expressed desire for this message to change their own husband’s behavior.

I felt a little envious about the message. The couple is so happy, if my husband changes when he gets to see the message then it would be so good.

Participant 19

Participants reported that men were also very involved in decision making for family planning methods; most commonly women explained that there were discussions between the couple regarding what methods to choose, but the final decision maker was more likely to be the husband.

I know my husband is very conscious and whatever he will decide, will also be my decision.

Participant 1

Women said their husbands would generally be supportive of them receiving messages on their phone, but some said that they would need their husband’s permission to receive them, or at least to inform him beforehand.

Yes, I am interested, but I need his permission.

Participant 2

If my husband knows about the matter beforehand then there is no problem. If my husband finds out that I have chosen to follow a method without his concern, then it will result in marital dispute. If he is in a good mood, he probably will say “you could’ve let me know at least.”

Participant 4

## Discussion

### Principal Findings

In this exploratory study, we asked women questions about their interest in contraceptive mHealth services and about their views on sample mHealth message content and modalities. Women strongly welcomed the idea of receiving messages about family planning on their mobile phones after their menstrual regulation and wanted the option of being able to talk to a call center counselor. Women reported that the type of information included in the sample messages would be helpful to them; in particular, they wanted information about the method of contraception they were using, as well as guidance on which methods they could use after the menstrual regulation and which were best suited to them.

Women liked the interactive content as it gave them control over the information they received, that is, they can select the additional information or services that are relevant to them. Reminders were seen as particularly useful for those women using short-term methods, as they knew that forgetting to use the method would have an impact on its effectiveness. In 2013, a pilot study tested the feasibility of using a simple, one-way SMS to send contraceptive reminders to menstrual regulation clients in Bangladesh, of which 93% (51/55) of women reported that the SMS helped them use their method correctly [25].

New information on family planning was seen as something valuable that could benefit not just the recipient but also other women in her family and networks. Their husbands' involvement in family planning decision making and use also appeared very important to women; women wanted to share the message content with their husbands and were interested in their husbands talking to counselors. Women also reacted very strongly to a positive example of a man helping his wife to use the contraceptive pill. As many women report that men make the final decision for family planning methods [26], it is important to consider how best to support women who want to engage more with their husbands on this topic.

mHealth interventions have been gaining popularity and one of the reasons for this is their potential for broad reach; yet, although disparities in mobile phone ownership are decreasing [27], they still exist. Poorer or less educated women have less access to mobile phones than urban women [15,28], and ownership is still higher among men than women (61.76% (1213/1964) versus 34.38% (1015/2952) [21].

Voice messages were favored over SMS, and most women found interactive voicemail easy to use. Women in our study suggested that rural and less educated women may have difficulty reading SMS messages, which is consistent with findings that literacy is lower among rural women and those in the lowest socioeconomic quintile [15]. Women found the Bengali words in English letters difficult to read and would have preferred Bengali script. Most phones in Bangladesh are not smartphones and lack the capability to support Bengali script. This may at least, in part, explain why among rural mobile phone users in Bangladesh only 49.87% (1111/2228) know that SMS messages can be received and sent, and only 36.62% (816/2228) read SMS messages [21]. Similar difficulties have been reported in Cambodia where pictures or audio messages were suggested as alternatives to SMS [29].

The findings from this exploratory study will be used to inform an mHealth intervention for postmenstrual regulation family planning in Bangladesh and may be used for development of similar interventions in the future. Including the target population in the development of mHealth messages can help to ensure that their views, preferences, and needs are taken into account. Voice messages may be preferential in terms of effort expectancy and ease of use for the women; however, information shared in an automated call has to be received at when it is delivered, rather than when the individual is ready as is the case with SMS or pull-type interventions such as call centers. The desire to have a number to call for information was expressed in similar studies in Bangladesh [25,30]. Women receiving mobile phone messages about reproductive health could be given the option of connecting to the call center.

Although women felt it would be better if the services were provided free of charge, many reported willingness to pay a fee if necessary. Women participating in feasibility research for Aponjon, a mobile phone intervention for expectant and new mothers, also reported not wanting to pay for mobile phone messages; however, the program used an income-based fee scale and found that those charged were willing to pay [30].

Social norms are known to be strong influencers of contraceptive behaviors. Women reported that confidentiality and privacy would not be a problem for them, and many reported that they wanted to share the messages, and information they contained, with other women so they could learn from them too. Women mentioned some people around them who they felt should not see the messages, meaning privacy could be a concern in some situations. Additionally, although women here did not seem very worried about privacy, other studies have found that women’s perception changed over the course of actually receiving the text messages, for example women in South Africa were more concerned about privacy after receiving the SMS intervention than they had been before [31].

### Limitations

This study aimed to inform the development of an intervention targeted at postmenstrual regulation family planning, and relevance to other interventions, contexts, or populations is likely to be limited. We made efforts to recruit participants from a range of socioeconomic backgrounds by recruiting women from clinics in both urban and peri-urban areas of Dhaka and Sylhet, but we did not recruit women from a more rural clinic as planned because of political violence at the time of data collection. Half of the participants in the sample were educated to secondary level or higher; it is likely that women in our sample have higher education and income levels, as well as more experience and greater fluency with technology than their counterparts in more rural areas of Bangladesh.

A further limitation is the use of convenience sampling, which is inherently biased as it is a nonprobability sampling technique. The views shared by respondents in this sample may not be representative of all women who are accessing menstrual regulation services and own mobile phones.

All participants in our sample had had an MVA procedure. Medical menstrual regulation clients may have differing needs in terms of follow-up information, at least in the short term. For example, they may be more focused on the menstrual regulation procedure than on family planning because their procedure is not yet complete. Bias could have been introduced during the sampling; providers may have been more inclined to ask certain women to participate, possibly those they felt were more approachable or who had been satisfied with their menstrual regulation procedure, who may in turn have been more likely to want to participate. Clients who were not feeling well after the menstrual regulation or were in a hurry may also have been missed.

A further limitation of the methodology is that women were asked to give their views on a hypothetical intervention: the example messages were shared during the interview at a time when the woman was somewhere quiet and had time to listen carefully without interruption. Responses to the content and length of messages and to questions about privacy may differ in a real-life intervention when the timing of messages delivery may not be so convenient.

### Conclusions

Overall, this study showed that this sample of Bangladeshi menstrual regulation clients were very interested in receiving information on their phones to support family planning use after their procedure, providing a solid foundation for the planned intervention. Women were most interested in learning more about the method of contraception they took after their menstrual regulation procedure and how to recover after their menstrual regulation procedures.

 Interactive voicemail was the preferred modality and would give women in our study the option to select the information most useful to them. This approach could satisfy both those who are keen to hear information about a range of methods and those who only want content about the method they are currently using. An interactive system could also include an option to be connected directly to a call center counselor. Drawbacks of voice messages are that they are harder to share and cannot be played when it is most convenient; interactive SMS could be offered as an alternative or in addition to voice messages for literate clients. Sharing information on a sensitive topic such as family planning by mobile phone has privacy implications, and this is an area that should be closely monitored when delivering interventions on this topic.

## Appendix

Multimedia Appendix 1 Interactive voice message format.

## Abbreviations

IUCD: intrauterine contraceptive device
mHealth: mobile health
MVA: manual vacuum aspiration
NGO: nongovernmental organization
RCT: randomized controlled trial
SMS: short message service
