# Supplementary figures and images for: Developing mHealth Messages to Promote Postmenstrual Regulation Contraceptive Use in Bangladesh: Participatory Interview Study
Source: JMIR Mhealth Uhealth. 2017 Dec 14;5(12):e174. doi: 10.2196/mhealth.6969 (PMC5754199; doi:10.2196/mhealth.6969)

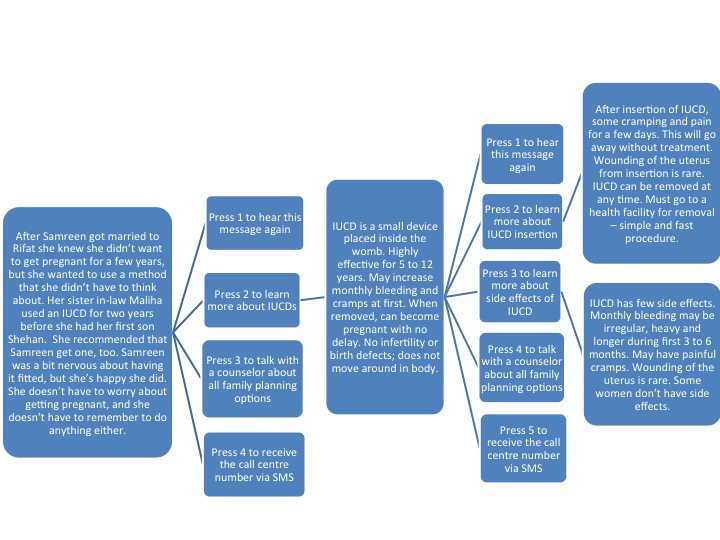

Supplement: Multimedia Appendix 1 [file mhealth_v5i12e174_app1.jpg]
